# Targeted therapy in pediatric central nervous system tumors: a review from the National Pediatric Cancer Foundation

**DOI:** 10.3389/fonc.2025.1504803

**Published:** 2025-02-28

**Authors:** Benjamin I. Siegel, Prabhumallikarjun Patil, Akul Prakash, Darren M. Klawinski, Eugene I. Hwang

**Affiliations:** ^1^ Brain Tumor Institute and Gilbert Family Neurofibromatosis Institute, Children’s National Hospital, Washington, DC, United States; ^2^ Division of Oncology, Children’s National Hospital, Washington, DC, United States; ^3^ Children’s Healthcare of Atlanta, Aflac Cancer Center, Atlanta, GA, United States; ^4^ Department of Pediatrics, Emory University School of Medicine, Atlanta, GA, United States; ^5^ New York University, New York, NY, United States; ^6^ Division of Hematology/Oncology, Nemours Children’s Health Jacksonville, Jacksonville, FL, United States

**Keywords:** neuro-oncology, targeted therapy, MAPK, mTOR, epigenetics, brain tumors, VEGF, receptor tyrosine kinase

## Abstract

Central nervous system tumors represent the leading cause of cancer-related mortality in children. Conventional therapies of surgery, radiation, and cytotoxic chemotherapy have insufficient efficacy for some pediatric CNS tumors and are associated with significant morbidity, prompting an ongoing need for novel treatment approaches. Identification of molecular alterations driving tumorigenesis has led to a rising interest in developing targeted therapies for these tumors. The present narrative review focuses on recent progress in targeted therapies for pediatric CNS tumors. We outline the key implicated cellular pathways, discuss candidate molecular therapies for targeting each pathway, and present an overview of the clinical trial landscape for targeted therapies in pediatric CNS tumors. We then discuss challenges and future directions for targeted therapy, including combinatorial approaches and real-time drug screening for personalized treatment planning.

## 1 Introduction

The core treatment modalities for pediatric central nervous system (CNS) tumors are surgery, radiation, and cytotoxic chemotherapy. While effective for some patients, these modalities are inadequate for many CNS tumor types and can cause significant morbidity. Recently, with better understanding of the underlying molecular drivers of pediatric cancer, targeted therapy has emerged as a promising alternative, or adjunct, to traditional cancer treatment. Targeted therapy aims to disrupt specific molecular pathways that drive tumor growth and progression. The underlying principle is that by targeting specific molecules involved in the growth and spread of cancer cells, on-target effects will increase and damage to healthy tissues will be attenuated.

In this narrative review, we outline the key pathways implicated in pediatric CNS tumors and evaluate specific targets for therapeutic intervention. Using these molecular pathways as a framework, we present a primer on the clinical trial landscape for targeted therapies in pediatric CNS tumors by surveying key completed (**Table 1**) and ongoing (**Table 2**) trials. Finally, we discuss innovative approaches to employing targeted therapy, including combinatorial regimens and real-time drug screening for personalized treatment planning.

**Table 1:** Summary of key completed trials using molecular targeted therapy in pediatric CNS tumors.

Study Identifiers	Tumor Type	Agent(s)	Molecular Target	Study Design	Sample	Outcome	Treatment-Related Toxicities
MAPK pathway							
NCT01338857 (1)	LGG	Sorafenib	Multi-kinase inhibitor (BRAF, VEGF, PDGFR)	Phase 2 open label trial for progressive pLGG	N=11	PD in 9 (82%) patients Median TTP 2.8 mo; enrollment terminated early	Any grade: Rash (75%), dry skin (33%), elevated ALT/AST (33%/42%), anorexia (25%), diarrhea (42%), lymphopenia (25%) Grade ≥3: Diarrhea (9%), transaminitis (18%), headache (9%), mucositis (9%), rash (18%)
NCT01677741 (2)	LGG	Dabrafenib	BRAF V600E	Phase 1/2a single arm, open-label trial for progressive, refractory, or recurrent pLGG with BRAFV600E mutation	N=32	ORR 44% 1-year PFS 85%	Any grade: Fatigue (35%), rash (31%), arthralgia (25%), vomiting (22%), headache (22%) Grade ≥3: Rash (9%), arthralgia (3%), hypotension (3%), DIC (3%)
NCT02684058 (3)	LGG	Dabrafenib/Trametinib	BRAF V600E (D), MEK1/2 (T)	Phase 2 randomized trial comparing D/T to C/VCR chemotherapy for first-line treatment of pLGG with BRAFV600E mutation	N=110: 73 D/T, 37 C/VCR	For D/T group: ORR 47%, clinical benefit (at least stable disease) in 86%; For C/VCR group: ORR 11%, clinical benefit in 46%	For D/T group: Any grade: Pyrexia (68%), headache (48%), vomiting/diarrhea (34%/29%), fatigue 32%), dry skin/rash (26%/19%) Grade ≥3: Pyrexia (8%), weight gain (7%), neutropenia (10%), increased ALT (5%)
NCT02684058 (4)	HGG	Dabrafenib/Trametinib	BRAF V600E (D), MEK1/2 (T)	Phase 2 trial for progressive or relapsed pHGG with BRAF V600E mutation	N=41	ORR: 56% Median duration of response: 22.2 mo Median OS: 32.8 mo	Any grade: Pyrexia (51%), headache (34%), dry skin/rash (32%/22%), vomiting/diarrhea (29%/24%) Grade ≥3: Headache (10%), vomiting/diarrhea (5%/2%), neutropenia (2%), rash (2%)
NCT01089101/PBTC029 (5)	LGG	Selumetinib	MEK1/2	Phase 2 trial for progressive, refractory, or recurrent pLGG with BRAF aberration (fusion or mutation) or NF1	BRAF group N=25, NF1 group N=25	BRAF group: PR in 36% 2-year PFS 70% NF1 group: PR in 40%, 2-year PFS 96%	Any grade: Elevated CPK (60%), anemia (56%), dry skin (56%), acneiform/maculopapular rash (58%/52%), vomiting/diarrhea (44%/54%), decreased ejection fraction (38%), peripheral edema (26%) Grade ≥3: Elevated CPK (10%), maculopapular rash (10%), diarrhea (4%), decreased ejection fraction (2%), headache (2%), gastric hemorrhage (2%)
NCT04775485/FIREFLY-1/PNOC026 (6)	LGG	Tovorafenib	BRAF	Phase 2 open label trial for relapsed/refractor pLGG with BRAF alteration (fusion or mutation, arm 1) or RAF-activating alteration (arm 2)	N=77 in primary analysis (arm 1)	ORR 51% by RAPNO criteria Clinical benefit rate (at least stable disease) 82%	Any grade: Hair color changes (76%), anemia (59%), elevated CPK (56%), fatigue (44%), vomiting (20%), hypophosphatemia (35%), maculopapular/acneiform rash (41%/30%), paronychia (24%), epistaxis (20%), decreased growth velocity (13%) Grade ≥3: Elevated CPK (12%), anemia (10%), maculopapular rash (8%), fatigue (4%), increased ALT (4%), decreased growth velocity (5%)
RTK							
NCT00042991 (7)	HGG	Gefitinib	EGFR	Phase I/II open label trial of gefitinib and irradiation for newly diagnosed pediatric gliomas	N=44 enrolled, 43 eligible and evaluable;	12- and 24-month PFS rates were 20.9% and 9.3% and OS 56.4% and 19.6%, respectively. 6 ORR	Any Grade: skin (42%), Gastrointestinal toxicity (42%), ocular toxicity (23%) Grade ≥ 3: Lymphopenia (21%), neutropenia (2%), Gastrointestinal toxicity (12%), infection (7%), pulmonary toxicity (5%), renal toxicity (2%), skin toxicity (2%), metabolic toxicity (2%)
NCT01644773 (8)	HGG	Dasatinib	PDFGRA	Phase I open label trial for recurrent/progressive high-grade and diffuse intrinsic pontine glioma	N=25	No objective radiologic responses	Any grade: Anemia (64%), neutropenia (17%), thrombocytopenia (8%), diarrhea (84%), Nausea/vomiting (60%), Transaminitis (36%), Hypoalbuminemia (68%), Hyponatremia (32%), Hypokalemia (44%), Hypophosphatemia (68%), Proteinuria (48%), Rash (40%), Fatigue (48%) Grade ≥ 3: Neutropenia (4%), diarrhea (8%), hyponatremia (8%), hypokalemia (8%), hypophosphatemia (16%), proteinuria (4%), rash (8%), fatigue (8%)
NCT03210714 (9–11)	HGG	Erdafitinib	FGFR	NCI-Children’s Oncology Group Pediatric Molecular Analysis for Therapy Choice (MATCH) Arm B evaluating FGFR inhibitor erdafitinib in patients with tumors harboring activitating FGFR alterations	Active, not recruiting N=6 with HGG	No objective radiologic responses	Any grade: Hyperphosphatemia, nail changes, nail infections
NCT02650401 (12)	HGG	Entrectinib	NTRK	Phase I/II open label trial of entrectinib in patients <22 with solid tumors with NTRK, ROS1, or ALK fusions	N=43 total patients N=16 with CNS Tumors and N=3 with HGG NOS and N=3 with GBM	50% ORR in CNS tumors	Any grade: Weight gain (49%), Anemia (40%), Creatinine inc. (40%), nausea (35%), constipation (30%), ALT inc. (28%), AST inc. (26%), Neutrophil count dec. (35%), White blood cell dec. (21%), Vomiting (21%) Grade ≥ 3: weight gain (16%), ALT inc. (5%), Neutrophil count dec (26%), White blood dec. (5%), Fracture (5%)
VEGF							
NCT00381797 (13)	LGG	Bevacizumab	VEGF	Phase II open-label trial of bevacizumab plus irinotecan in children with recurrent LGG	N=35	6-month and 2-year PFS rates 85% and 47.8%	Any grade: Hypertension (69%), Fatigue (66%), Epistaxis (51%), Proteinuria (43%), Grade ≥ 3: proteinuria (9%), Avascular necrosis of lunate bone (3%)
NCT00271609 (14)	HGG	Bevacizumab	VEGF	Phase II open label trial of single-agent bevacizumab in patients with recurrent anaplastic glioma	N=31	Median OS 12 mos with median PFS 2.93 mos ORR 67% (20 PR)	Any grade: hypertension (32%), proteinuria (29%), epistaxis (26%), headache (23%), thrombocytopenia (23%) Grade ≥ 3: hypertension (16%), proteinuria (3%), headache (3%), hypophosphatemia (6%), rash (3%), thrombus (6%), hyperuricemia (3%), retinopathy (3%), hyponatremia (3%)
PI3K/mTOR pathway							
NCT00789828/EXIST-1 (15)	SEGA	Everolimus	mTOR	Phase 3 double-blind, placebo-controlled randomized trial for children and adults with TSC and SEGA	Everolimus, N=78; Placebo, N=39	ORR 35% in everolimus group (vs 0% in placebo)	Any grade: Mouth ulceration/stomatitis (32%/31%), pyrexia (22%), vomiting/diarrhea (17%/13%), rash (12%) Grade ≥3: Stomatitis (8%), pyrexia (6%)
NCT00782626/POETIC (16)	LGG	Everolimus	mTOR	Phase 2 open-label, single-arm trial for progressive pLGG	N=23	ORR 13%; Clinical benefit rate (at least stable disease) 52% 2-year PFS 26%	Grade ≥3: Mucositis (12%), elevated ALT/AST (6%), pneumonitis (6%), neutropenia (6%)
NCT05009992/PNOC022 (17)	DMG	Paxalisib/ ONC201	PI3K (paxalisib)	Phase 2 open label trial for DMG pre-radiation (Cohort 1), post-radiation (Cohort 2), or at progression (Cohort 3)	N=132 total, 33 Cohort 1, 69 Cohort 2, 30 Cohort 3	Median OS from diagnosis 13.2 mo in Cohort 1 and 15.8 in Cohort 2; Median OS from progression 8.8 mo in Cohort 3	Grade ≥3: Maculopapular rash (9%), mucositis (6%), colitis (5%), hyperglycemia (7%)
Cell cycle alterations							
NCT02607124 (18)	DMG	Ribociclib	CDK4/6	Phase 1/2 open-label trial for newly-diagnosed DMG post-radiation	N=10	Median OS from diagnosis: 16.1 mo 1-year OS: 89%	Any grade: Vomiting (50%), elevated ALT (40%), thrombocytopenia (40%), fatigue (30%), anemia (30%) Grade ≥3: Leukopenia (70%), anemia (10%), hypokalemia (20%), hyponatremia (10%), hypophosphatemia (10%)
Epigenetic alterations							
NCT02717455/PBTC-047 (19)	DIPG	Panobinostat	HDAC	Phase 1 dose escalation trial for progressive DIPG (Stratum 1) or newly-diagnosed DIPG/DMG post-radiation (Stratum 2)	Stratum 1: N=19 Stratum 2: N=34	Median OS from diagnosis: 11.8 mo (Stratum 2)	Any grade (dose level 1): Thrombocytopenia (62%), Increased ALT (46%), hypertension (23%), fatigue (23%), anemia (23%) DLT: Observed in 10 of 51 (20%) patients overall: neutropenia (10%), thrombocytopenia (10%), nausea (2%), increased ALT (2%)
NCT03416530/ONC201-014 and NCT03134131/ONC201-018 (20)	DMG	ONC201	Cellular metabolism	Pooled analysis of two phase 1/2 trials, including only newly-diagnosed H3K27M-DMG post-radiation	N=35	Median OS from diagnosis: 21.7 months, compared to 12.0 months in historical controls	For ONC201-014: TEAE, Grade ≥3: hemiparesis (14%), abdominal pain (5%), respiratory disorder (9%) (21)
Hedgehog pathway							
NCT01125800 (22)	Varied	Sonidegib	SMO	Phase 1 dose escalation trial for children and adults with relapsed/refractory medulloblastoma or other tumors suspected to have Hh pathway activation	Pediatric MB: N=21 Adult MB: N=16 Other pediatric tumors: N=21	ORR among pediatric tumors: 2/60 (3%); Among Hh-activated tumors: ORR 5/10 (50%) Among Hh-negative tumors: 0 responders	For RP2D, 680 mg/m2 Any grade: Elevated CPK (23%), myalgia (23%), vomiting (14%) Grade ≥3: Elevated CPK (9%) Note: growth plate closure observed in 3 pediatric patients

LGG, low grade glioma; HGG, high grade glioma; GBM, glioblastoma, SEGA, subependymal giant cell astrocytoma; DMG, diffuse midline glioma; DIPG, diffuse intrinsic pontine glioma; PD, progressive disease; TTP, time to progression; TEAE, treatment-emergent adverse event; ORR, objective response rate; PFS, progression-free survival; OS, overall survival; NF1, neurofibromatosis type 1; TSC, tuberous sclerosis; DLT, dose-limiting toxicity.

**Table 2:** Ongoing clinical trials using molecular targeted therapy in pediatric CNS tumors.

Study Identifiers	Tumor Type	Agent(s)	Target of Molecular Agent(s)	Study Design	Primary Endpoint(s)	Status*
MAPK pathway						
NCT03919071/ ACNS1731 (23)	BRAF V600-mutant HGG	Dabrafenib/trametinib after radiation	BRAF, MEK	Phase 2, single arm	EFS	Recruiting
NCT03871257/ ACNS1831 (24)	Previously untreated NF1-associated LGG	Experimental: selumetinib Active comparator: carboplatin/vincristine	MEK	Phase 3, randomized, parallel assignment	EFS, visual acuity	Recruiting
NCT04166409/ ACNS1833 (25)	Previously untreated LGG in patients without NF1	Experimental: selumetinib Active comparator: carboplatin/vincristine	MEK	Phase 3, randomized, parallel assignment	EFS	Recruiting
NCT04576117/ ACNS1931 (26)	Recurrent or progressive LGG	Experimental: selumetinib with vinblastine Active comparator: selumetinib monotherapy	MEK	Phase 3, randomized, parallel assignment	MTD (selumetinib/vinblastine), EFS	Recruiting
NCT04201457/ PBTC055 (27)	Recurrent or progressive LGG or HGG with BRAF alteration	BRAF V600E+: Dabrafenib, trametinib, hydroxychloroquine BRAF alteration or NF1: trametinib, hydroxychloroquine	BRAF, MEK	Phase 1/2	Phase 1: MTD, PK Phase 2: Sustained ORR	Recruiting
NCT05465174/ PNOC029 (28)	Newly diagnosed or recurrent craniopharyngioma	Tovorafenib, nivolumab, either as monotherapy or in combination	RAF	Phase 2, randomized 1:1:1, parallel assignment	PFS, QOL	Recruiting
NCT05286788/ CONNECT2108 (29)	Newly diagnosed or recurrent/progressive adamantinomatous craniopharyngioma	Binimetinib	MEK	Phase 2, single intervention	Sustained ORR	Recruiting
NCT04923126/ SJ901 (30)	Previously untreated (during phase 2 only) or progressive/recurrent (phase 1 and 2) LGG	Mirdametinib	MEK	Phase 1/2, single intervention	Phase 1: MTD, safety/toxicity, PK Phase 2: ORR, stabilization rate	Recruiting
NCT05566795/ FIREFLY-2 (31)	Previously untreated LGG with activating RAF alteration	Experimental: tovorafenib Active comparator: standard-of-care chemotherapy (per investigator choice)	RAF	Phase 3, randomized, parallel assignment	ORR	Recruiting
NCT03363217/ TRAM-01 (32)	Progressive/refractory CNS glioma	Trametinib	MEK	Phase 2, single intervention	ORR	Active, not recruiting
RTK						
NCT04655404/ CONNECT1903 (33)	Newly-diagnosed HGG with NTRK fusion	Larotrectinib	NTRK	Phase 1	Disease control rate (CR/PR/SD), safety/toxicity, PK	Recruiting
NCT06528691/ GLBOTRK (34)	Newly-diagnosed CNS tumor with NTRK1/2/3 or ROS1 gene fusion in patients <3 yo	Entrectinib	NTRK, ROS1	Phase 2, single intervention	ORR	Recruiting
NCT04094610 (35)	Solid or CNS tumor with ROS1 alteration or NTRK1/2/3 fusion	Repotrectinib	NTRK, ROS1	Phase 1/2, single intervention	Phase 1: DLT rate, RP2D Phase 2: ORR	Recruiting
NCT04773782/ ROVER (36)	Relapsed/refractory solid and CNS tumors with PDGFRA or KIT alterations	Avapritinib	PDGFRA	Phase 1/2, single arm	Phase 1: RP2D Phase 2: ORR	Active, not recruiting
NCT03598244/ PBTC049 (37)	Recurrent, progressive or refractory MB, HGG, DIPG; or other CNS tumor with MET aberration	Volitinib	MET	Phase 1, single arm	MTD, safety/toxicity, PK	Recruiting
Epigenetic alterations						
NCT04732065/ PNOC023 (38)	Newly diagnosed, recurrent/progressive DMG or other recurrent CNS tumors	ONC206	Cellular metabolism	Phase 1	MTD, Number with DLT	Recruiting
NCT05580562/ ACTION (39)	Newly-diagnosed DMG	ONC201	Cellular metabolism	Phase 3, randomized double-blind, placebo controlled	OS, PFS	Recruiting
Multiple/varied pathways						
NCT04485559/ PNOC021 (40)	Recurrent/progressive LGG or HGG (recurrent/progressive or newly diagnosed)	Trametinib, everolimus	MEK, MTOR	Phase 1	MTD, PK, safety/toxicity	Recruiting
NCT05057702/ PNOC027 (41)	Relapsed MB	Various, based on real-time drug screening and molecular tumor board recommendation	Various	Single-arm pilot feasibility trial	Feasibility	Recruiting
NCT05009992/ PNOC022 (42)	Newly diagnosed, recurrent/progressive DMG	ONC201, paxalisib, other targeted therapies	Cellular metabolism, PI3K	Platform trial	PFS, OS, number requiring dose modification	Recruiting
NCT05843253/TarGeT-A (43)	Newly diagnosed DMG or other HGG with cell cycle or PI3K/mTOR pathway alteration	Ribociclib, everolimus	CDK4/6, mTOR	Phase 2, single intervention	PFS, OS, MTD, safety/toxicity	Recruiting

*As of January 2025, per ClinicalTrials.gov.

CR, complete response; DLT, dose limiting toxicity; DMG, diffuse midline glioma; EFS, event-free survival; HGG, high-grade glioma; LGG, low-grade glioma; MB, medulloblastoma; MTD, maximum tolerated dose; NF1, neurofibromatosis type 1; ORR, objective response rate; OS, overall survival; PFS, progression-free survival; PK, pharmacokinetics; PR, partial response; QOL, quality of life; SD, stable disease.

## 2 MAPK pathway alterations

**Figure 1:**
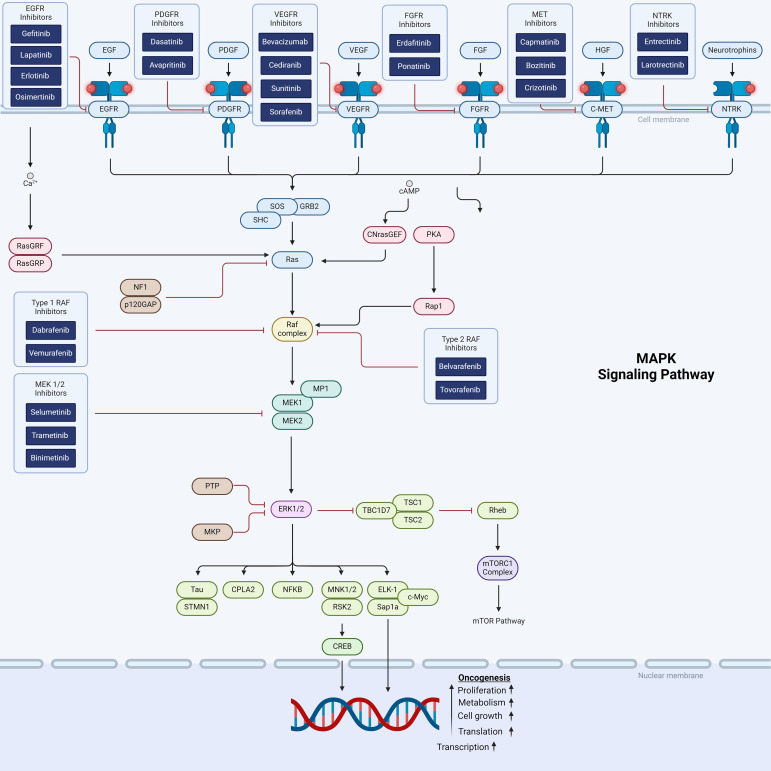
Schematic representation of the MAPK and RTK cellular signaling showing targets for therapeutic intervention.

The mitogen-activated protein kinase (MAPK) signaling cascade is one of the first described and most extensively studied pathways in cell biology. MAPK signaling is complex with multiple upstream and downstream interactions with other major pathways involved in cell proliferation, differentiation and other metabolic signals supporting tumor sustenance and growth (**Figure 1**). Direct downstream RAS signaling is composed of RAS–RAF–MAPK kinase (MEK) – ERK. In physiologic conditions, this cascade is activated by receptor tyrosine kinase (RTK), which triggers the GTPase-dependent RAS. Neurofibromin-1 (NF1) is a negative regulator of RAS, which in turn activates RAF kinase. RAF is a primary mediator of the MAPK pathway and is responsible for the sequential activation of downstream targets MEK1/2 and the transcription factor ERK 1/2. ERK1/2 transcriptionally regulates genes involved in proliferation and cell survival, including cAMP response element–binding protein, as well as the transcriptional regulator Myc-like (c-Myc) and nuclear factor kappa B (NFKB) (44).

The MAPK pathway is implicated in the vast majority of pediatric low grade gliomas (pLGGs), to the point that pLGG is thought to act as single pathway disease (45). Over 85% of pLGGs exhibit molecular aberrations of RAF, which ultimately upregulate the MAPK pathway (46, 47). Activating alterations of BRAF can occur as point mutations, in-frame deletions, or fusions with other kinases. The two most common alterations are the BRAFV600E mutation, caused by nucleotide transversion resulting in the substitution of valine (V) with glutamic acid (E) at position 600 (i.e., V600E point mutation) and tandem duplication resulting in BRAF-KIAA 1549 fusion (48–50). Amongst pLGGs, pleomorphic xanthoastrocytomas and gangliogliomas histologies are more commonly are associated with BRAFV600E, whereas pilocytic astrocytoma mostly harbor fusion of BRAF: KIAA1549 (51, 52). Although most data is in pLGG, 5-10% of pediatric high-grade gliomas also have MAPK pathway alterations (53). MAPK pathway activation has also been implicated in adamantinomatous craniopharyngioma (54), providing a target for multiple ongoing craniopharyngioma trials (**Table 2**).

### 2.1 BRAF V600E

The BRAF V600E mutation is seen in 15-20% of pLGG and is associated with higher resistance to chemotherapy and progression to higher grade malignancy (47, 51, 55, 56). Type-1 RAF inhibitors stabilize RAF in its active confirmation and block its catalytic activity. Dabrafenib is a Type-1 RAF inhibitor originally approved for advanced BRAFV600E-positive melanoma. In pediatric oncology, dabrafenib was first applied as a monotherapy in a phase I/IIa multicenter, open-label study in pediatric patients with advanced BRAF V600E mutation–positive solid tumors (NCT01677741). The drug was well-tolerated overall, with the most common adverse events being mild to moderate mucocutaneous and gastrointestinal toxicity. For those with pLGG, meaningful clinical benefit was noted, with an objective response rate of 44% and a 1-year estimated progression-free survival rate of 85% by independent review (2, 57). Similar safety and efficacy signals were noted in an early-phase trial of vemurafenib, another Type-1 RAF inihibitor (58, 59). A phase II study for vemurafenib in pLGG is ongoing (NCT01748149) (58).

The combination of BRAF inhibition with downstream MEK inhibition was first shown to be of clinical value in adults with non-small cell lung cancer (NSCLC) and anaplastic thyroid cancer (60–64). Based on the adult experience, a phase 1/2 trial in children with relapsed or refractory BRAFv600E pLGG were treated with either trametinib monotherapy or dual therapy with trametinib and dabrafenib. The combination group had a PR rate of 25%, compared to 15% in the monotherapy group (65). A subsequent phase 2 trial with combination of dabrafenib and trametinib was conducted for BRAFV600E pLGG in the upfront setting (3). This randomized trial compared the dabrafenib/trametinib combination to traditional chemotherapy with carboplatin/vincristine The targeted therapy group had an overall response rate of 47%, compared to 11% in the chemotherapy group. Additionally, clinical benefit (at least stable disease for >24 weeks) was observed in 86% of patients receiving dabrafenib/trametinib, compared to 46% in those receiving carboplatin/vincristine. These results led to FDA approval of dabrafenib/trametinib for upfront treatment of BRAF V600E-mutant pLGG (66). A rollover trial NCT03975829 aims to study long-term effects of therapy with dabrafenib, trametinib, or a combination of both drugs in pediatric patients.

The BRAF V600E mutation also occurs in 5-10% pHGGs (53). Data from case reports and retrospective reviews indicate that dabrafenib may be effective in relapsed and refractory pHGG with BRAFV600E mutation (4, 67). An ongoing phase 2 COG trial NCT03919071 aims to now study upfront targeted therapy, combining dabrafenib and trametinib after focal radiation for BRAF V600E–mutant pHGG.

### 2.2 BRAF-KIAA fusion

The BRAF-KIAA fusion causes dysfunction of the BRAF N-terminal regulatory domain, which normally regulates downstream RAS/MAPK signaling. Approximately one third of pLGG exhibit the BRAF-KIAA fusion (45, 49). MEK1/2 inhibitors inhibit the MAPK pathway downstream of RAS and RAF (**Figure 1**), and have shown clinical activity in BRAF-altered pLGG.

Selumetinib has shown clinical benefit in phase -1 and phase -2 clinical trials of recurrent and refractory pediatric low-grade gliomas with BRAF aberrations (5, 68, 69). In a key phase 2 trial run by the PBTC consortium, selumetinib showed an objective response rate of 40% for BRAF-altered progressive or recurrent pLGG and a 2-year PFS of 70%. By comparison, the landmark COG trial evaluating carboplatin/vincristine in the upfront setting for pLGG reported an objective response rate of 46% and a 2-year PFS of 87% (5, 70). Two ongoing phase III studies aim to study standard chemotherapy to upfront selumetinib in patients with newly diagnosed pLGG in patients with or without NF1, respectively (NCT03871257 and NCT04166409).

Other MEK inhibitors studied in pediatric CNS tumors include trametinib and binimetinib. In addition to its role in upfront therapy BRAFV600E pLGG, trametinib is currently being examined in other pLGG subtypes. An ongoing clinical trial NCT03363217/TRAM-01 aims to study trametinib as a monotherapy in a basket trial involving four groups of progressive tumors (KIAA1540-BRAF fusion, NF1-associated plexiform neurofibromas, NF1-associated other gliomas, and other MAPK-ERK pathway–activated gliomas) (71). Interim analysis on 53 evaluable patients reported 25 (47%) with at least a minor response and 48 (91%) with at least stable disease (72). In a pre-clinical model, binimietinb demonstrated superior CNS penetration compared to other MEK inhibitors, prompting a phase 2 trial evaluating its efficacy in progressive pLGG (72). Of 28 evaluable patients with BRAF fusion, 12 (43%) had a partial response and 26 (93%) had at least stable disease. However, significant toxicity was observed, with 22% discontinuing due to toxicity and 49% requiring dose-reduction.

Early experience with type -1 RAF inhibitors in LGG with BRAF fusions demonstrated paradoxical pathway activation through RAF dimerization, resulting in rapid tumor progression (1). Subsequently, Type 2 RAF inhibitors have been developed including tovorafenib, which has high CNS penetrance and does not paradoxically activate RAS. The FIREFLY-1 study (NCT04775485), a phase 2 trial of tovorafenib in progressive or recurrent BRAF-altered pLGG, reported an overall response rate of 51% by RAPNO criteria and a clinical benefit rate (defined as at least stable disease) of 82% (6). The FIREFLY-1 study led to FDA approval for tovorafenib for relapsed/refractory pLGG in 2024. FIREFLY-2 (NCT05566795), a follow-up phase 3 trial evaluating tovarafenib in for pLGG in the upfront setting, is underway (9).

## 3 Receptor tyrosine kinase alterations

Receptor tyrosine kinases (RTKs) are a family of cell surface proteins which act as receptors for growth factors, hormones, cytokines, neurotrophic factors, and other extracellular signaling molecules. This family of receptors is divided into subfamilies including epidermal growth factor receptor (EGFR), platelet-derived growth factor receptor (PDGFR), fibroblast growth factor receptor (FGFR), insulin and insulin-like growth factor receptor (IGFR), vascular endothelial growth factor receptor (VEGFR), and hepatocyte growth factor receptor (HGFR/C-MET) (10, 73–77). Once activated, RTKs initiate a signal cascade primarily through two downstream pathways: RAS/MAPK/ERK and RAS/PI3K/AKT. Ultimately, these pathways result in cell proliferation, invasiveness, survival, and angiogenesis. Aberrations in RTKs are commonly found in both pediatric high- and low-grade gliomas and are therefore promising therapeutic targets for treatment (10, 73–77). The most common genetic RTK alterations in these tumors occur in the EGFR family, followed by altered PDGFR and MET tyrosine kinase pathways. Multiple RTK inhibitors have and are currently being developed and evaluated in clinical trials (10, 73–77).

### 3.1 EGFR

Mutations in EGFR are the most common RTK aberrations in glioblastoma (GBM) and thus are an important therapeutic target. Both amplification and mutations in EGFR have been detected and are implicated in the pathogenesis and resistance to treatment of GBM cells (10, 73–77). To date, most studies with EGFR-tyrosine kinase inhibitors (TKI) or antibodies have shown limited efficacy likely due to poor CNS penetration of these drugs. Phase I and II clinical trials with first generation EGFR inhibitors gefitinib, lapatinib, and erlotinib have demonstrated marginal therapeutic response in primary and recurrent GBM. While second generation EGFR inhibitors did show response in GBM xenograft models, they showed limited activity in clinical trials of recurrent GBM. Osimertinib, a third generation EGFR inhibitor, is currently approved as first-line treatment for CNS metastatic disease in non-small cell lung cancer with EGFR mutations secondary to its high CNS penetrance and preclinical and clinical activity (74, 78). Preclinical data have demonstrated that osimertinib can reach high concentrations in the CNS and can be effective against EGFR mutated glioblastoma (78). This has prompted the use of osimertinib alone or in addition to conventional chemotherapy in patients with EGFR mutated GBM including the current study NCT03732352 (74, 79, 80). There are various reports of clinical experience using osimertinib in combination with bevacizumab or temozolomide which have not only shown the feasibility of combining EFGR inhibition with other therapy, but also a prognostic benefit in the upfront and recurrent setting following radiation (79, 81).

### 3.2 PDGFR

Dysregulation of PDGFR signaling contributes to oncogenesis in high-grade gliomas and have been associated with worse prognosis. The most common are mutations leading to amplification of PDGFRA in approximately 15% of pediatric high-grade gliomas and lead to significantly higher PDFGRA expression (10, 73–77, 82). Interestingly, an analysis of 290 pHGG reported that the mutation itself, rather than PDGFRA amplification, was of prognostic significance (82). The drug dasatinib has demonstrated high PDGFR inhibition and CNS penetration, but when used as a single agent in recurrent adult GBM it did not show efficacy (77, 82). In preclinical studies, the PDGFRA inhibitor avapritinib demonstrated significant decrease in tumor growth and improved survival in mouse models of pediatric PDGFRA mutated H3K27M DMG. Subsequently, the drug was used in 8 pediatric and young adult patients with PDGFRA-altered diffuse midline glioma (DMG) or other high-grade glioma. There were no significant acute toxicities within the cohort and 50% of patients exhibited a radiographic response (83). These findings have led to the Phase 1/2 study of avapritinib (ROVER) in pediatric patients with relapsed/refractory solid tumors dependent on KIT or PDGFRA signaling (84).

### 3.3 FGFR

Abnormal expression of fibroblast growth factor receptors (FGFR) is the second-most common molecular aberration in sporadic pLGG (behind BRAF). In particular, FGFR1 has been shown to be disrupted through either point mutations or copy number variations and mutations (10, 73–77). The drug erdafitinib has demonstrated preclinical and clinical activity in pediatric gliomas (85) harboring FGFR mutations and has been investigated in a phase I trial for solid tumors including GBM showing partial responses. Another FGFR inhibitor ponatinib has also demonstrated favorable CNS penetration on pharmacokinetic analysis (75, 77).

### 3.4 NTRK

The neurotrophic tropomyosin kinase (NTRK) genes (NTRK-1, NTRK-2, and NTRK-3) are located on chromosomes 1 (1q22), 9 (9q22), and 15 (15q25) and code for the receptor tyrosine kinase proteins TRK-A, TRK-B, and TRK-C, respectively. Activation of these receptors leads to downstream signaling cascades including Ras/MAPK, phospholipase C-γ (PLC-γ), and PI3-K and are involved in normal neurodevelopment (86). NTRK gene fusions are frequently reported in both pediatric and adult tumor populations and lead to constitutively activated TRK and tumorigenesis. More than 50 fusions have been described; however, the general structural rearrangement is preserved with the overall result of a chimeric protein keeping the NTRK tyrosine kinase domain ligand-independent (86). Aberrations involving the NTRK genes have been found in both pLGG and pHGG, including infant high-grade glioma (45, 53, 56, 86, 87). The prevalence of NTRK fusions has been reported in as high as 40% in infant high-grade gliomas, 10% in non-brain stem pediatric high-grade gliomas, 4% in diffuse intrinsic pontine gliomas, and < 1% of pediatric low-grade glioma (12, 86–88). Entrectinib was the first drug developed for NTRK fusionsand has good CNS penetrance. Entrectinib is further appealing for use infantile hemispheric high grade glioma because it also targets ALK and ROS1 fusions, which, in addition to NTRK fusions, are commonly seen in this tumor type (87). Entrectinib was tested in the phase I and phase II STARTRK trials and showed promising results in pediatric and adult CNS tumors harboring NTRK fusions with an ORR of 50% (12, 86), and is being further evaluated in a phase 2 trial of children less than 3 years old with CNS tumors harboring NTRK or ROS1 fusion (34). Larotrectinib was developed as a highly specific NTRK inhibitor with good CNS penetrance and antitumor activity in patients with NTRK fused CNS malignancy. Larotrectinib has been evaluated in the pediatric clinical trial SCOUT and the adult and pediatric trial NAVIGATE, which both included patients with primary CNS tumors. A pooled analysis of these trials showed that 82% of patients with measurable disease had tumor shrinkage with a 12-month PFS of 56% and favorable safety profile (86, 89, 90). A recent multicenter retrospective cohort study included 16 pediatric patients with NTRK-fusion gliomas treated with larotectinib and demonstrated an objective response rate in 11 (69%) patients (91). An early-phase clinical trial using larotrectinib in the upfront setting for pediatric HGG with NTRK fusion is underway (NCT04655404) (92).

### 3.5 MET

Mesenchymal-epithelial transition (MET) is an RTK that contributes to growth and angiogenesis of pediatric high-grade glioma as it is expressed and activated in tumor cells and vascular endothelial cells resulting in cellular proliferation and invasion (10, 73–77). Genetic alterations in MET have been associated with poor prognosis in GBM (74). Inhibition of MET with capmatinib was studied in a phase II trial in adult GBM and showed no clear activity while the MET inhibitor bozitinib was tested in 18 pediatric patients with recurrent high-grade glioma with partial response seen in only 2 patients (77). Another inhibitor of MET crizotinib is being studied in combination with temozolomide and radiotherapy for newly diagnosed GBM (NCT02270034) and with dasatinib in pediatric patients with diffuse intrinsic pontine glioma and high-grade glioma (NCT01644773) (74).

## 4 VEGF

High-grade gliomas have structurally and functionally abnormal vasculature. Beginning in the mid-1990s, studies showed that inhibition of vascular endothelial growth factor (VEGF) expression in GBM reduced vasculature formation and suppressed tumor growth (10, 74). The most widely used drug to target VEGF is the humanized monoclonal anti-angiogenic antibody bevacizumab, which is the first FDA-approved targeted treatment for recurrent GBM. Bevacizumab has showed improved progression-free survival (PFS) in GBM, but has not shown benefit to overall survival when used alone (14, 74). In pediatric neuro-oncology, bevacizumab has shown efficacy in combination with irinotecan in progressive or recurrent low-grade glioma (13, 93). Bevacizumab may also have specific application for optic pathway gliomas, where it has demonstrated a favorable association with visual outcomes (94, 95). The VEGFR inhibitor cediranib was investigated in a phase II clinical trial as monotherapy in recurrent GBM showing improved radiographic response at 6 months, but no overall survival benefit. The drug sunitinib, which targets PDGFRA/B in addition to VEGFR, showed preclinical promise, but it did not show improvement of PFS in patients with recurrent GBM in a phase II clinical trial (74, 77).

## 5 PI3K/mTOR pathway alterations

**Figure 2:**
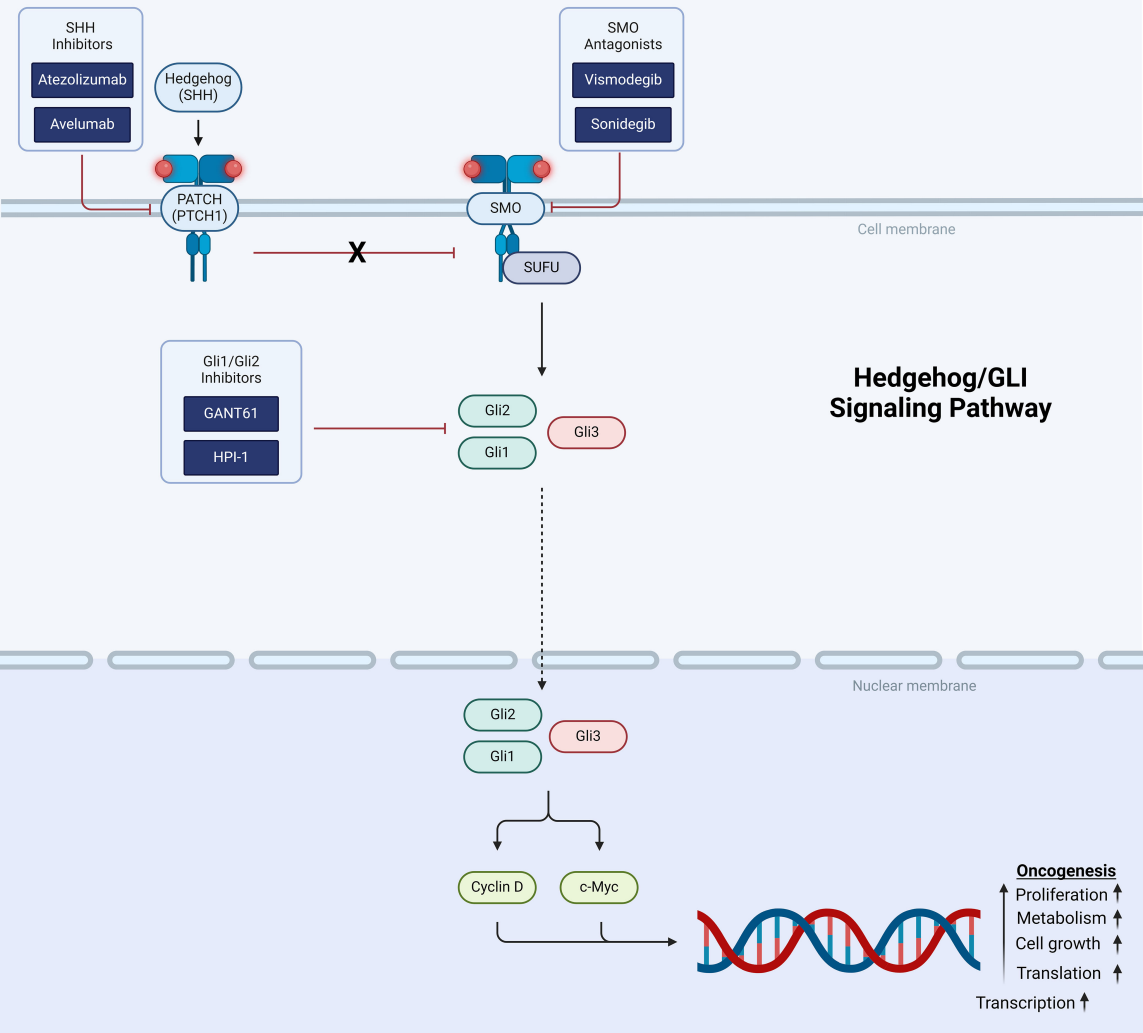
Schematic representation of the PI3K/mTOR signaling pathway showing target points for therapeutic intervention.

The PI3K/mTOR pathway is a signal transduction pathway involved in cell growth and proliferation and is another critical target for tumor-directed therapy in pediatric CNS tumors. Once activated at the cell membrane, phosphatidylinositol 3-kinase (PI3K) leads to the accumulation of PIP3, which in turn leads to phosphorylation and activation of Akt (96) (**Figure 2**). Activated Akt inhibits the GTPase tuberin-hamartin (TSC) complex. Inactivation of the TSC complex disinhibits mTOR, initiating downstream promotion of cell proliferation and survival. Activation of PI3K, a critical entry point to the PI3K/mTOR pathway, can be achieved through activation of transmembrane proteins including RTKs (eg, FGFR) and insulin-like growth factor 1 receptor (IGF-1R). PI3K can also be activated directly by RAS, leading to signficant crosstalk between the PI3K/mTOR and MAPK pathways. Alterations in the PI3K/mTOR pathway are ubiquitous in human cancer and have been specifically implicated in pediatric high- and low-grade glioma (97).

Most efforts to target the pathway are focused on inhibiting PI3K or mTOR. For example, for subependymal giant cell astrocytoma (SEGA) associated with the tumor predisposition syndrome tuberous sclerosis, the mTOR inhibitor everolimus is now FDA-approved in the upfront setting based on a landmark phase 3 clinical trial showing its efficacy (15). Attempts to apply mTOR inhibitor monotherapy to other tumor types have been less successful. A phase 2 trial of everolimus monotherapy in recurrent or progressive pediatric LGG showed a partial response rate in only 2 of 23 (13%) patients (16), and a separate study demonstrated that PI3k/mTOR pathway activation did not correlate with response (98). Similarly low response rates were observed in a trial of recurrent NF1-associated LGG treated with everolimus (99).

The crosstalk between the MAPK and PI3K/mTOR pathways creates an appealing opportunity for combinatorial treatment approaches. The combination of everolimus with the MEK inhibitor trametinib is currently being evaluated in the clinical trial NCT04485559 for patients with pediatric gliomas (either low or high grade).

Alterations in the PI3K/Akt/mTOR pathway have also been identified as molecular drivers in H3K27-altered diffuse midline glioma (53). The PI3K inhibitor paxalisib has demonstrated efficacy in pre-clinical DMG models when used in combination with other agents (100, 101), prompting its incorporation into an ongoing clinical trial with using an adaptive platform design (PNOC022/NCT05009992). Preliminary data from this trial indicate that the combination of paxalisib and ONC201 is generally well-tolerated, with the most common treatment-related adverse events being myelosuppression, rash, mucositis, and colitis (17).

## 6 Cell cycle alterations

Cyclin-dependent kinases (CDKs) are protein complexes involved in cell cycle regulation. CDK4 and CDK6 are of specific relevance in cancer and promote the transition from G1 to S cell cycle phases through the phosphorylation of retinoblastoma protein (Rb) (102). Inhibition of CDK4/6 by the tumor suppressor protein P16 causes G1 cell arrest by inhibiting CDK4/6 activity (103). Homozygous deletions of *CDKN2A*, the gene that codes for P16, result in unregulated cell division through disinhibition of CDK4/6 and have been implicated in multiple cancer types, including in BRAF V600E gliomas. Indeed, *CDKN2A* deletion is an independent negative predictor of outcome in V600E-mutant pLGG (51). *CDKN2A* homozygous deletions are also common in IDH-WT HGGs, can occur independently of BRAF alterations, and are associated with a worse clinical outcome (104).

CDK4/6 inhibitors are a class of small-molecule drugs designed to recapitulate the physiologic function of P16 that is lost with CDKN2A deletion, thereby promoting cell cycle arrest. Ribociclib is a CDK4/6 inhibitor with good CNS penetration (105). In a phase I/II clinical trial for DIPG, ribociclib was well-tolerated and associated with increased necrotic tumor volume but did not provide significant clinical benefit (18). A putative explanation for failure of CDK4/6 inhibitor monotherapy is reversal of cell-cycle arrest when the drug is withdrawn. This has led to efforts for combinatorial therapies as such as the TarGeT-A trial, which combines ribociclib with the mTOR inhibitor everolimus (**Table 2**).

## 7 Epigenetic alterations

Epigenetic changes involve modification to gene expression, rather than alteration to genes themselves. A complex interplay between DNA and histone modification results in a dynamic switching of genes “on” and “off”, as well as modulation of the level of gene expression (106). In the nucleus, DNA is packaged as chromatin. The basic structural unit of chromatin is the nucleosome, which is a coil of DNA wrapped around a histone core. The histone core is an octamer made up of 8 proteins, 2 each of H2A, H2B, H3, and H4. Each histone protein has an amino acid tail, which is relevant for gene expression, and are abundant in lysine and arginine. H3 is of specific relevance to pediatric brain tumors.

One of the ways that transcription is regulated is through modulating the wrapping and unwrapping of DNA around histone octamers. This is done through modification of the DNA itself, through methylation, and through modification of the histone amino acid tails - byacetylation and methylation. Histone acetylation results in a relative negative charge. DNA is also negatively charged, so when there is histone acetylation, there is loosening of the DNA coil around the histone, facilitating transcription. In turn, histone methylation creates a docking site for chromatin-associated proteins. Histone methylation can result in activated or repressed chromatin, depending on the site. Methylation of specific lysine residues on the amino acid tails of H3 and H4, including H3K9, H3K27, and H4K20 result in transcriptional suppression. Of particular interest in CNS tumors is trimethylation of H3K27, abbreviated H3K27me3, which results in transcriptional suppression by Polycomb Repressive Complex 2 (PRC2). By contrast, methylation of other lysine residues, including H3K4, H3K36, and H3K79 results in transcriptional activation.

In contrast to adult HGG, histone modifications are seen in about 80% of pHGG. The most common somatic alteration seen in pHGG are variants resulting in the presence of a methionine (M) instead of a lysine (K) at position 27 on the amino acid tail of histone H3, H3K27M. This results in chromatin remodeling and loss of trimethylation of H3K27 (H3K27me3) with subsequent transcriptional activation and tumorigenesis. The H3K27M mutation is a hallmark molecular finding in diffuse midline glioma (DMG) (53). DMG is associated with dismal clinical outcomes and multiple chemotherapeutic regimens have been evaluated without significant improvement on survival. In the past decade, there has been rising interest in the small molecule ONC201/dordaviprone, which showed signs of efficacy in adult patients with progressive H3K27M DMG (107). Recently, evidence has emerged indicating that the anti-tumor effect of ONC201 in DIPG is through disruption of the TCA cycle within mitochondria, leading to an inhibitory effect on histone lysine demethylases and increase in genomic H3K27me3 (20). A pooled analysis of two clinical trials (NCT03416530/ONC201-014 and NCT03134131/ONC201-018) evaluating ONC201 in non-recurrent H3K27M-mutant DMG identified a modest improvement in median overall survival compared to historical controls (OS 21.7 months vs 12.0 months, respectively) (20). ACTION (NCT05580562) is an ongoing randomized trial comparing ONC201 to placebo in newly-diagnosed H3K27M-mutant DMG (39). Another histone alteration involves replacement of glycine (G) with valine (V) or arginine (R) at position 34 on histone H3.3. G34R and G34V mutants result in transcriptional activation and are commonly seen in pediatric-type hemispheric high grade gliomas (53).

### 7.1 HDAC

Histone deacetylases (HDACs) are enzymes which catalyze the removal of acetyl functional groups from histone proteins, ultimately resulting in gene inactivation. Inhibition of HDACs prevents deacetylation and therefore results in gene activation through chromatin opening. Panobinostat is a non-selective HDAC inhibitor that is FDA-approved for the treatment of multiple myeloma. Panobinostat has demonstrated efficacy in orthotopic xenograft models of DIPG (108). However, its application in humans has been limited by significant dose-limiting toxicity (particularly myelosuppression) (19) and limited CNS penetration (109). To achieve adequate target exposure at tolerable doses of panobinostat, novel approaches using convection-enhanced delivery (CED) are being explored (110). Fimepinostat is another HDAC inhibitor which, when used in combination with gemcitabine, demonstrated a synergistic anti-tumor effect in an orthotopic H3K27M DIPG xenograft model and represents a potential therapeutic strategy for future trials (111).

### 7.2 EZH2

Epigenetic alterations are also seen in embryonal tumors, including medulloblastoma and atypical teratoid/rhabdoid tumor (ATRT). In both medulloblastoma and ATRT, there is overexpression of EZH2, an enzyme component of PRC2 involved in the methylation of H3K27 (106, 112). Overexpression of EZH2 results in widespread trimethylation of H3K27 and ultimately tumorigenesis through decreased tumor suppressor gene activity (113). Tazemetostat is a selective EZH2 inhibitor FDA-approved in epithelioid sarcoma. Early experience in ATRT has been promising; in a case series of 4 pediatric patients with ATRT treated with tazemetostat in the upfront setting following resection and conventional chemotherapy, a 2 had a PR and 2 had a CR, with 3 of 4 patients alive at last follow-up (OS 30-34 mo) (7).

## 8 Hedgehog pathway alterations

**Figure 3:**
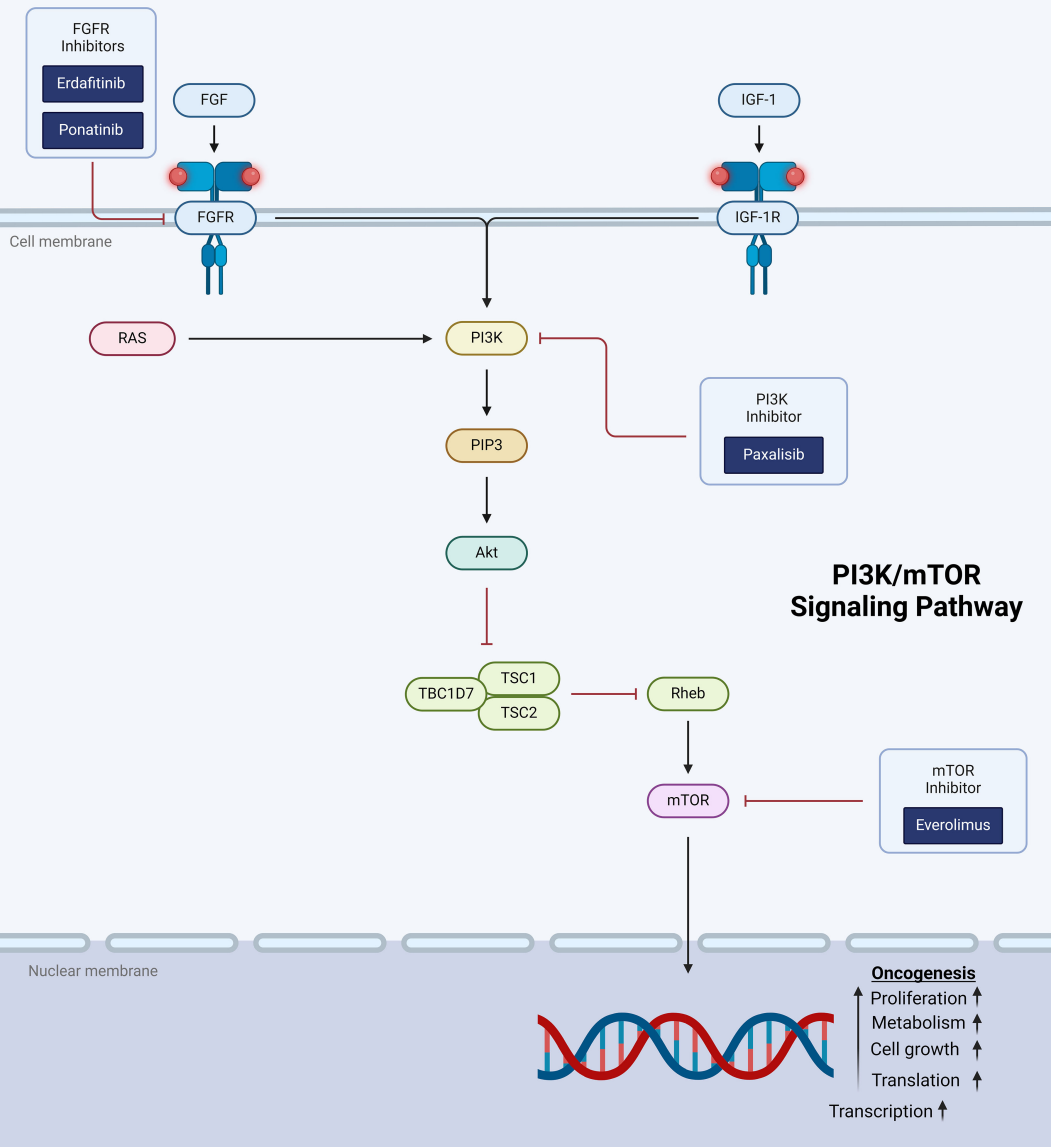
Schematic representation of the hedgehog/GLI signaling pathway showing targets for therapeutic intervention.

The Hedgehog/Glioma-associated oncogene homolog (HH/GLI) pathway controls various processes during embryonic development including cerebellar maturation and tissue regeneration. Sonic hedgehog (SHH), a component of the HH/GLI pathway, is critical for normal cerebellar development, but constitutive activation of SHH signaling results in tumorigenesis (8, 11, 114, 115). Hedgehog pathway signaling involves the 12 pass-transmembrane receptor, PATCH (PTCH1) and when bound releases its inhibition of smoothened (SMO), a protein that activates the downstream portion of the pathway by binding to the cell fusion inhibitor called suppressor of fused (SUFU) and induces nuclear translocation of activators Gli1 and Gli2 and a repressor Gli3 (**Figure 3**). The Gli proteins regulate the expression of downstream targets including Cyclin D and MYC involved in cell survival, proliferation, and differentiation (8, 11, 114). Mutations in this pathway drive the initiation and progression of the SHH subtype of medulloblastoma as well as other solid tumors. Therefore, various agents have been developed targeting SHH, SMO and Gli1 and Gli 2 (8, 11, 114).

SHH inhibitors atezolizumab and avelumab prevent the binding of SHH to PTCH1 and are being studied in the preclinical setting. Vismodegib and sonidegib are SMO antagonists and were approved by the FDA in 2012 and 2015, respectively, for the treatment of advanced or metastatic basal cell carcinoma (8, 11, 114). In initial an early Phase I study with vismodegib only 1 of 3 patients with SHH activated medulloblastoma had antitumor response. This has been corroborated subsequent SMO inhibitor trials (22, 116), and may be explained by intra-group heterogeneity within the SHH subgroup. Vismodegib was also found to induce irreversible growth plate fusion with prolonged exposure (117). The drugs GANT61 and HPI-1 target Gli1 and Gli2 but have not been tested in medulloblastoma (8, 114). Resistance to SMO inhibition was first described in 2009 and the patient with medulloblastoma had mutations in the SMO gene. It has since been described that mutations in SMO lead to both *de novo* and acquired resistance and neither vismodegib or sonidegib are effective in certain mutations (114). Hyperactivation of GLI has been shown to lead to chemoresistance and radiation resistance in multiple cancers including medulloblastoma. This increase in the pathway ultimately suppresses the anti-tumor response from the immune system (8, 11, 114, 115, 118, 119).

## 9 Discussion

The development of targeted therapies has been driven by a deepening understanding of the molecular underpinnings of pediatric CNS tumors. This expanding knowledge base has enabled the identification of specific molecular targets for therapeutic intervention. Nevertheless, success has been uneven across the pediatric neuro-oncology landscape. The greatest advances have been seen in low grade glioma, a largely single-pathway disease and currently the only pediatric CNS tumor with FDA-approved targeted therapies – dabrafenib/trametinib for upfront treatment of BRAF V600E mutant LGG and tovorafenib for BRAF-altered LGG in the recurrent setting. A common theme in this review is that monotherapy with targeted agents is rarely sufficient for durable treatment response. As illustrated in **Table 2**, several current clinical trials are using combinatorial approaches to achieve the sweet spot of optimizing efficacy while mitigating toxicity. These include employing multiple targeted agents addressing discrete implicated pathways (eg, everolimus/trametinib for LGG and HGG, NCT04485559), combining conventional treatment modalities with targeted agents, and using molecular therapy along with immunotherapy to create a synergistic anti-tumor effect.

Other trials are using a personalized medicine approach via a “molecular tumor board” that incorporates a patient’s individual tumor molecular profile to determine a treatment plan. In addition to providing rationale treatment recommendations for a given tumor’s molecular profile, this centralized approach has appealing equity implications by improving access to specialized care (120). The PNOC003/NCT02274987 trial used a molecular tumor board to recommend personalized treatment regimens for children with DIPG (121). The multidisciplinary tumor board considered clinical and genomic data before providing a consensus recommendation of up to 4 FDA-approved drugs to be included in the treatment regimen within 21 days of surgery (122). Thirty-eight participants were enrolled, 28 of whom were evaluable by the tumor board (123). Nineteen (68%) patients followed tumor board treatment recommendations, supporting the feasibility of the approach. There was no difference in survival for those who followed tumor board recommendations compared to those who did not. Nevertheless, experience from this trial informed the development of PNOC022, on ongoing platform trial that includes an arm for molecularly-guided combinatorial molecular therapy for DMG.

Another innovative trial design uses real-time drug screening with live tumor tissue to provide the molecular tumor board with more robust information in developing an individualized treatment plan (124). For example, the ongoing trial PNOC027/NCT05057702 conducts high-throughput drug screening on freshly isolated tumor cells of children with relapsed medulloblastoma. The platform evaluates responses to 232 clinically-available compounds. A recent preliminary report of 9 patients enrolled on the study demonstrated the feasibility of the approach: 8 of the 9 patients successfully completed real-time drugs screening, with a median turnaround time of 7 days from sample receipt (125).

While outside the scope of this review, there is also considerable interest in optimizing delivery of targeted therapies beyond traditional oral, intravenous, or intrathecal routes. For example, convection enhanced delivery, low intensity focused ultrasound, and nanoparticle-based therapies have been employed as tools to circumvent the blood-brain barrier and modulate the immune microenvironment (126–129).

An underlying impetus for developing targeted therapy, in addition to increasing treatment efficacy, is avoiding the systemic toxicities seen in traditional cytotoxic chemotherapy. With notable exceptions (eg, bevacizumab), targeted therapies have the additional benefit of availability of oral formulations, sparing patients the need for durable central venous access or hospital admissions for drug administration. Many targeted therapies are also less immunosuppressive compared to cytotoxic chemotherapies, decreasing the risk for serious infections. Nevertheless, as presented in **Table 1**, these drugs are not without adverse effects. Grade 1/2 “nuisance” toxicities including mucocutaneous and gastrointestinal effects are common, and serious effects on cardiac, liver, and bone marrow function have been observed. Additionally, due to their novelty, long-term effects are not well characterized.

Although promising, molecular targeted therapy remains in its infancy. Questions remain regarding the optimal sequence and duration of therapies. Strategies to address “rebound” phenomena, in which early growth is observed after therapy discontinuation, remain unsettled. Finally, while immediate toxicity profiles are generally favorable compared to traditional cytotoxic chemotherapy, long-term effects of targeted therapies on growth, fertility, and cognitive function are not yet known.
